# Safety and Tolerability of the Gut Bacterium *Phascolarctobacterium faecium* DSM 32890

**DOI:** 10.3390/nu18030498

**Published:** 2026-02-02

**Authors:** Maria Tamayo, Veronica Tolosa-Enguis, Blanca Alabadi, Marta Olivares, Sergio Romera, Leticia Orti, Elisabet Terrado, Alejandra Flor Duro, Carlos Morillas, Pilar Codoñer, José T. Real, Yolanda Sanz

**Affiliations:** 1 Microbiome Innovation in Nutrition and Health Research Unit (INNOBIOME), Institute of Agrochemistry and Food Technology, Spanish National Research Council (IATA-CSIC), 46980 Valencia, Spain; 2 INCLIVA Biomedical Research Institute, 46010 Valencia, Spain; 3 CIBER de Diabetes y Enfermedades Metabólicas Asociadas (CIBERDEM), 28029 Madrid, Spain; 4 Endocrinology and Nutrition Service, University Hospital Doctor Peset, 46017 Valencia, Spain; 5 Endocrinology and Nutrition Service, Hospital Clínico Universitario de Valencia, 46010 Valencia, Spain; 6 Department of Medicine, University of Valencia, 46010 Valencia, Spain

**Keywords:** *Phascolarctobacterium faecium*, safety and tolerability, gut microbiome, obesity, type 2 diabetes

## Abstract

Background/Objectives: The prevalence of the commensal gut bacterium species, *Phascolarctobacterium faecium*, has been associated with normal weight in humans. Preclinical evidence suggests that the strain *P. faecium* DSM 32890 exerts beneficial effects on metabolic and immune function in diet-induced obesity. Herein, we aimed to evaluate the safety and tolerability of this strain in a preclinical study and a pilot interventional trial in humans. Methods: A repeated-dose oral toxicity study of 28 days was performed in Wistar rats (male and female), during which adverse signs and clinical outcomes were assessed, along with histological, hematologic, biochemical, and immune markers. Subsequently, a pilot human intervention trial was conducted, including 20 participants (11 overweight and 9 normal weight) who received *P. faecium* DSM 32890 daily for 15 days. Body composition, dietary intake, physical activity, clinical data, perceived health, gastrointestinal symptoms, and blood analyses were assessed to determine tolerability and identify potential adverse effects. Results: In rats, the administration of the bacterium did not cause behavioral, physiological, histologic, immune, or biochemical alterations. In humans, there was no evidence of adverse effects on general health, hematological and biochemical profiles, bowel habits, or gastrointestinal symptoms. Overweight participants experienced reductions in flatulence and nausea after the intervention. Conclusions: The consumption of *P. faecium* DSM 32890 did not raise safety concerns and was well tolerated in rats and humans. The findings represent a step forward in the path toward future, longer-term studies to explore the potential efficacy.

## 1. Introduction

Obesity is a complex, multifactorial condition frequently associated with hypertension, hyperglycemia, dyslipidemia, elevated uric acid, and inflammation, which have a profound negative impact on subjects’ health and quality of life. Obesity also acts as the umbrella for a cluster of chronic lifestyle-related diseases (type-2 diabetes, cardiovascular disease, etc.), which represent the main causes of mortality. Altogether, this places substantial pressure on healthcare systems and imposes a significant economic burden on national budgets [1].

Western dietary patterns, sedentary behavior, and inadequate sleep habits have contributed to a doubling of obesity rates over the past 30 years [1]. Furthermore, these lifestyle factors, especially diet, impact the composition and function of the gut microbiota [2]. The gut microbiota interacts with dietary components and the host’s enteroendocrine and immune pathways, thereby regulating energy homeostasis. Diet-induced gut microbiota disruption can, in turn, impair microbial functional roles, leading to metabolic dysregulation and the development of obesity and chronic low-grade inflammation. Therefore, restoring and enriching the gut microbiota with missing microbes is being investigated as a strategy to help prevent obesity and its associated comorbidities [3].

The gut commensal species *Phascolarctobacterium faecium*, an anaerobic, Gram-negative bacterium that produces short-chain fatty acids (SCFAs), has been recently linked to a lean phenotype in humans [4]. In the same study, the strain *P. faecium* DSM 32890 has been shown to counteract diet-induced obesity by reprogramming innate immunity, thereby reducing macrophage-induced inflammation and improving glucose tolerance in mice [4]. These findings suggest that this *P. faecium* strain could represent a potential novel next-generation probiotic for preventing and mitigating obesity and associated metabolic conditions.

The present study aimed to comprehensively assess the safety profile and tolerability of *P. faecium* DSM 32890 through a repeated-dose toxicity study in rats and a pilot human intervention trial, providing the necessary foundation for future larger efficacy trials.

## 2. Materials and Methods

### 2.1. Bacterial Culture Conditions and Quantification

*P. faecium* DSM 32890 was isolated from the fecal sample of a healthy donor, as previously reported [5]. For in vivo assays, *P. faecium* was cultured under anaerobic conditions at 37 °C for 36 h in a modified PYG medium (recipe 104 from the German type culture collection—DSMZ, Stöckheim, Germany), in which glucose was replaced with sodium succinate (8 g/L) as the primary carbon source. After incubation, bacterial cultures were centrifuged, washed with phosphate-buffered saline (PBS) supplemented with 0.05% L-cysteine (Sigma, St. Louis, MO, USA), and the resulting pellets were resuspended in PBS supplemented with 0.05% L-cysteine and 20% glycerol, then stored at −80 °C until use.

*Bifidobacterium longum* CECT 30763 was grown in commercial MRS medium (Scharlau; ref 02-135-500, Barcelona, Spain) and was incubated for 24 h at 37 °C under anaerobic conditions (80% N_2_, 10% CO_2_, and 10% H_2_). Afterward, cultures were centrifuged, and the resulting pellets were resuspended in PBS containing 0.05% L-cysteine and 20% glycerol and stored at −80 °C until use.

Flow cytometry and serial dilutions plated on modified PYG or MRS agar were used to determine colony-forming units (CFUs) for the in vivo assays in rats.

*P. faecium* DSM 32890 and a placebo were produced by SAS NeoBioSys (Issoire, France) for the intervention trial in humans. The total number of cells was quantified by flow cytometry, 4.6 × 10^10^ CFU/g.

### 2.2. Animal Experiment Design

**Figure 1:**
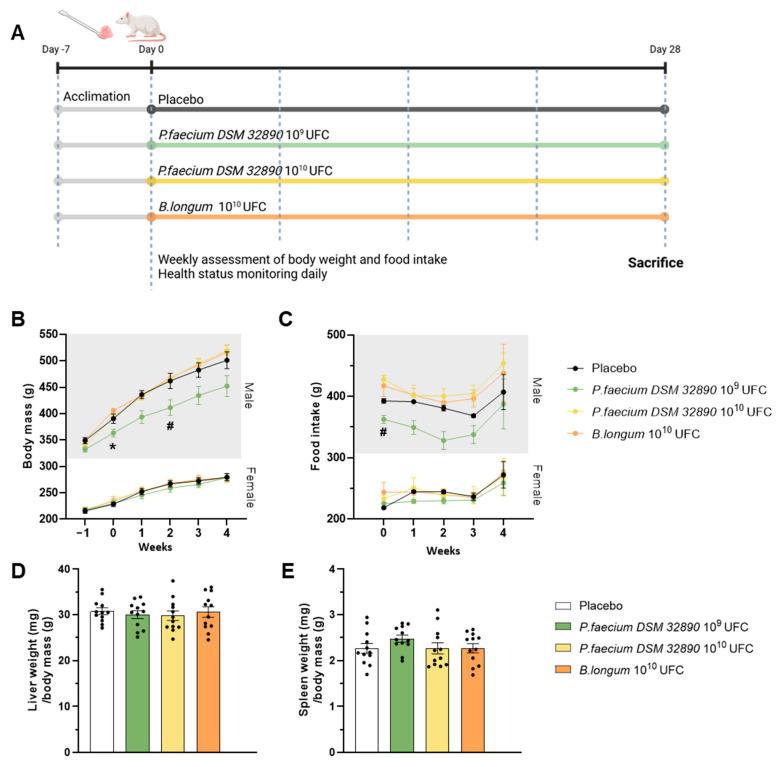
Design of the repeated-dose oral toxicity study and macroscopic examinations. (**A**) Experimental design. (**B**) Body weight evolution in male and female rats, and (**C**) food intake in both sexes. (**D**) Liver and (**E**) spleen weight normalized to body mass. Data are expressed as mean ± standard deviation. UFC per dose. Two-way ANOVA: * *p* < 0.05 *P. faecium* 10^9^ UFC vs. *P. faecium* 10^10^ UFC and *B. longum*; # *p* < 0.05 *P. faecium* 10^9^ UFC vs. *P. faecium* 10^10^ UFC. Figure 1A was created with BioRender.com.

Figure 1A represents a scheme of the experimental design. A total of 48 eight-week-old Wistar rats (Janvier Labs) were randomly assigned to four groups (n = 12/group; 6 males and 6 females) and housed in pairs by sex under controlled environmental conditions (23 °C, 12 h light/dark cycle, 40–50% humidity). After a 7-day acclimation period, microbiota composition was homogenized by mixing the beading of the different cages as previously described [6]. Animals received oral doses of bacteria or vehicle for 28 days as follows: (1) control vehicle; (2) *P. faecium* DSM 32890 at 1 × 10^9^ CFU (2.5 × 10^9^/kg); (3) *P. faecium* DSM 32890 at 1 × 10^10^ CFU (2.5 × 10^10^/kg); and (4) *B. longum* CECT 30763 at 1 × 10^10^ CFU (2.5 × 10^10^/kg). Bacteria were administered with a small piece of sweet jelly (4–5% sucrose) used as a vehicle. Throughout the experimental period, rats were examined daily for general health and signs of toxicity, including changes in skin, fur, eyes, mucous membranes, secretions, excretions, gait, posture, response to handling, and abnormal behavior. Body weight and food intake were recorded weekly.

At the end of the treatment period, the animals were fasted for 4 h and anaesthetized with a mixture of isoflurane and oxygen (5% for induction and 2% for maintenance). Once the absence of the pedal reflex was confirmed, blood was collected by cardiac puncture in K3-EDTA tubes (Microvette^®^ 500 EDTA K3, Sarstedt AG & Co., Nümbrecht, Germany) for hematological analysis and in heparinized tubes (Microvette^®^ 500 lithium heparin, Sarstedt AG & Co., Nümbrecht, Germany) for biochemical determinations. Following blood collection, animals were euthanized, and the liver, spleen, mesenteric lymph nodes (MLNs), and small and large intestines were dissected, processed, and stored appropriately for subsequent analyses.

Animal procedures complied with the European Union Directive 2010/63/EU on the protection of animals used for scientific purposes. They were approved by the Animal Experimentation Ethics Committee (CEEA) of the Prince Felipe Research Center Foundation. The study was evaluated and authorized by the ethical committee and the competent authority (Conselleria de Agricultura, Ganadería y Pesca of the Generalitat Valenciana (Authorization code: 2023-VSC-PEA-0232 type 2). The experimental protocol generally followed the recommendations of the Commission Regulation (EC) No 440/2008, which establishes test methods under the REACH for repeated-dose (28 days) oral toxicity testing [7] and the methodology used in similar toxicological assessments [8,9].

### 2.3. Hematological and Biochemical Analyses

Blood analyses were conducted at the Biomedical Imaging and Metabolomics Section of the Central Service of Support to Research (SCSIE, University of Valencia, Valencia, Spain). Hematological analysis included: White blood cell counts reported both as percentages (%) relative to total leukocytes and as absolute concentrations (10^9^/L), including total white blood cells (WBC), neutrophils (NEU), lymphocytes (LYM), monocytes (MON), eosinophils (EOS), and basophils (BAS); red blood cell counts (RBC), hemoglobin (HGB), hematocrit (HCT), mean corpuscular volume (MCV), mean corpuscular hemoglobin (MCH), mean corpuscular hemoglobin concentration (MCHC), red cell distribution width expressed as the coefficient of variation (RDW-CV), platelet count (PLT), and mean platelet volume (MPV). Biochemical parameters included glucose (GLU), total cholesterol (CHOL), alkaline phosphatase (ALP), alanine aminotransferase (ALT), total bilirubin (TBIL), total protein (TP), albumin (ALB), globulin (GLOB), creatinine (CREA), urea (UREA), amylase (AMY), lipase (LIPA), phosphorus (PHOS), calcium (C-Ca), blood urea nitrogen (BUN), albumin/globulin ratio (A/G), and BUN/creatinine ratio (B/C).

### 2.4. Bacterial Translocation Analyses

The blood samples and MLNs collected during euthanasia were immediately placed in anaerobic jars to preserve bacterial viability for the assessment of bacterial translocation. MLN samples were homogenized in PBS supplemented with 0.05% L-cysteine under anaerobic conditions, and blood samples were centrifuged to obtain serum. Both preparations were plated on selective media: modified PYG and MRS agar for analyzing the growth of *P. faecium* or *B. longum*, respectively. Plates were incubated anaerobically at 37 °C for 48 h. After incubation, CFUs were counted, and representative colonies were subjected to molecular identification using strain-specific primers for both strains (Supplementary Table S1). Individual colonies were resuspended in PBS, and PCR amplification was performed using the KAPA HiFi HotStart ReadyMix PCR Kit (Roche Sequencing Solutions, Basel, Switzerland) according to the manufacturer’s instructions.

### 2.5. Gene Expression Analysis

Total RNA was isolated from jejunum and colon tissues using the NucleoSpin RNA Mini Kit (Ref. 740955.250, MACHEREY-NAGEL GmbH & Co. KG, Dueren, Germany) following the manufacturer’s protocol. RNA concentration and purity were determined with a NanoDrop spectrophotometer (Thermo Scientific Inc., Bremen, Germany). Complementary DNA (cDNA) was synthesized from 1 µg of total RNA using the High-Capacity cDNA Reverse Transcription Kit (Ref. 4368814, Thermo Scientific Inc., Bremen, Germany). Quantitative real-time PCR was performed on a LightCycler^®^ 480 instrument (Roche Diagnostics S.L., Spain) in 20 µL reaction volumes containing SYBR Green Master Mix (Ref. 04707516001, Roche Diagnostics S.L., Barcelona, Spain). Expression levels were normalized to the endogenous reference gene Rplp2, and relative quantification was calculated using the 2^−ΔΔCt^ method. Primer sequences are listed in Supplementary Table S1.

### 2.6. Determination of Total IgA and Cytokine Concentrations

For IgA quantification, approximately 50 mg of cecal content were placed in Lysing Matrix D tubes (MP Biomedicals, Madrid, Spain) containing 1.4 mm zirconium silicate beads and diluted 1:10 in PBS (1×). Samples were vortexed for 30 s and centrifuged at 700 rpm for 5 min at 4 °C. The supernatants were collected, diluted 1:50, and analyzed by ELISA according to the manufacturer’s instructions. Absorbance was read at 450 nm using a spectrophotometer.

For cytokine quantification, proteins were extracted from jejunum samples, quantified by the Bradford method, and measured using a CLARIOstar Plus spectrophotometer (BMG LABTECH, Ortenberg, Germany),. Protein extracts were loaded into the multiplex plate (MILLIPLEX MAP Rat Cytokine/Chemokine Magnetic Bead Panel; Ref. RECYTMAG-65K, Merck, Darmstadt, Germany) and incubated with assay buffer for 10 min, followed by overnight incubation with magnetic beads at 4 °C. The next day, detection antibodies and streptavidin–phycoerythrin were sequentially added. Finally, Drive Fluid was incorporated, and cytokine concentrations were quantified using a MAGPIX^®^ instrument (Thermo Fisher Scientific Inc, Waltham, MA, USA) with xPONENT^®^ software (v4.3, Luminex Corp., Austin, TX, USA). Cytokines measured included interleukin (IL)-1β, IL-10, interferon gamma (IFNγ), and tumor necrosis factor (TNF).

### 2.7. Histological Analysis

Colon tissues were excised, rinsed with PBS, and fixed in 4% paraformaldehyde (PFA) in PBS (pH 7.4) for 48 h at room temperature, followed by storage in 0.5% PFA in PBS (pH 7.4). Samples were dehydrated, embedded in paraffin, sectioned at 3 µm thickness, and stained with hematoxylin–eosin for histological evaluation. Bright-field images (three colon sections per rat) were captured using a Nikon Eclipse 90i microscope equipped with a DS-5Mc 5-megapixel digital color camera (Nikon Corporation, Tokyo, Japan). Observations were performed under halogen illumination using a CFI Plan Fluor 4× objective (MRH00040) with an optical zoom of 0.8×. The length of the Lieberkühn crypts (10 per section) and the number of goblet cells were quantified manually using NIS-Elements BR 3.2 (Nikon Corporation, Tokyo, Japan) following standardized procedures to ensure reproducibility.

### 2.8. Intervention Trial in Humans

An interventional trial was conducted to evaluate the safety and tolerability of *P. faecium* DSM 32890. The study was registered at ClinicalTrials.gov (NCT07285317, 1 February 2024) and approved by the Ethics Committees of University Hospital Dr Peset of Valencia (CEIm:60-22) and of the Spanish National Research Council (CSIC) (217/2023).

A total of 20 adult volunteers from the Valencian Community (Spain) were enrolled in the study. Recruitment was conducted through the research group’s website and social media. Interested individuals completed an online questionnaire and were then appointed to confirm eligibility. Inclusion criteria include: adult individuals (men and women) aged 18–65 years, stable body weight and dietary habits during the previous three months, and providing written informed consent. Two groups were identified according to body mass index (BMI) and metabolic status: (1) a normal-weight (NW) group (BMI 18–25 kg/m^2^) and (2) an overweight/obese (OW) group (BMI > 25 kg/m^2^) presenting altered glucose homeostasis, defined by elevated fasting glucose, HOMA-IR, or fasting insulin levels. Exclusion criteria included gastrointestinal disorders, immunodeficiency, eating disorders, antibiotic use within 1 month before the start of the study, chronic antidiabetic therapy, substance abuse, restrictive diets, or any condition deemed inappropriate for participation by the clinician.

Participants consumed four capsules containing 250 mg of the bacterium once daily for 15 consecutive days, for a total daily dose of 1 g of *P. faecium* DSM 32890 (4.6 × 10^10^ CFU). Stool and blood samples were collected before and after the intervention. Blood was drawn after at least 8 h of fasting. Participants also completed self-reported questionnaires covering sociodemographic information, gastrointestinal symptoms, bowel habits, general health (PROMIS) [10], physical activity (IPAQ) [11], and diet (PREDIMED-FFQ) [12]. Whole-body dual-energy X-ray absorptiometry (DEXA) and anthropometric measurements were used to assess body composition in the OW group. DEXA, anthropometric measurements, and blood analyses were performed at the Endocrinology and Nutrition Unit of Hospital Clínico Universitario of Valencia, Spain.

### 2.9. Pre-Processing and Sequencing of Rat Fecal Samples

Genomic DNA was extracted from 100 mg of rat fecal material using the QIAmp Fast DNA Stool Mini Kit (No. 51604, Qiagen, Germany)/OJO EN EL LAB Tenemos “QIAmp PowerFecal Pro DNA Kit (No. 51804, Qiagen, Germany) according to the manufacturer’s instructions, with an additional mechanical disruption step to improve yield. This step consisted of bead beating in microcentrifuge tubes containing 0.1 mm Mini-BeadBeater glass beads (No. 11079101, BioSpec, Bartlesville, OK, USA). DNA quantity was measured fluorometrically using a Qubit fluorometer (No. Q32854, Thermo Fisher Scientific, Waltham, MA, USA), and purity was evaluated by the 260/280 absorbance ratio with a NanoDrop spectrophotometer (Thermo Fisher Scientific). DNA extracts were stored at –80 °C until sequencing.

Sequencing was performed using the Illumina MiSeq platform with the 16S rRNA V3–V4 primer set (2 × 275 bp paired-end) [13] and the Nextera XT v2 Index Kit for multiplexing at the Institute of Parasitology and Biomedicine “López-Neyra” (IPBLN-CSIC, Granada, Spain). Each sample yielded an average of 140,361 raw reads. Demultiplexing was conducted with USEARCH v9.2.64 [14]. Reads trimming, filtering, and merging (R1/R2) were followed by multi-step quality assessment using FastQC v0.12 [15] and MultiQC v1.14 [16]. Further processing was carried out through the DADA2 R package [17]. Reads shorter than 250 bp were excluded. Taxonomic classification of amplicon sequence variants (ASVs) was performed against the SILVA v138.1 database [18], incorporating updated nomenclature in accordance with the International Code of Nomenclature for Prokaryotes. Low-prevalence and non-bacterial ASVs were filtered using the Phyloseq R package v1.48 [19].

### 2.10. Microbiota Diversity Analysis

The phyloseq R package [19] was used to address the group differences across the sample level (alpha diversity) and the community level (beta diversity). Alpha diversity indexes included the Observed taxa abundance, Chao1, Inverse Simpson, and Shannon indexes to capture the specific microbial richness of the samples, the potential loss of rare taxa due to sequencing and trimming, and the uniformity of taxa distribution. Beta-diversity was examined through Principal Coordinates Analysis (PCoA) using Bray–Curtis (abundance-based) and Jaccard (presence/absence-based) distances to evaluate group differences.

To assess taxon-specific effects, we used the MaAsLin2 R package to test for differential abundance of ASVs between treatment groups and the placebo. Relative abundances were analyzed using linear models with Total Sum Scaling (TSS) normalization and a logarithmic (LOG) transformation, while adjusting for sex and filtering taxa with a prevalence < 0.15. Significant taxa were identified using Benjamini–Hochberg False Discovery Rate (FDR)-corrected q-values ≤ 0.05.

### 2.11. Statistical Analysis

Statistical analysis was performed mainly using GraphPad Prism 10 (Graph Pad Software Inc., San Diego, CA, USA). First, the normality of data distribution was assessed with the D’Agostino–Pearson omnibus test. According to the distribution and experimental design, statistical comparisons were performed using paired or unpaired *t*-tests, the Wilcoxon rank-sum test, one-way or two-way ANOVA followed by Bonferroni’s multiple-comparison test, or the Kruskal–Wallis test, as appropriate. Categorical variables were evaluated using the Chi-square test. Statistical significance was defined as *p* ≤ 0.05. Data are expressed as mean ± standard deviation (SD) for continuous variables and as n (%) for categorical variables.

## 3. Results

### 3.1. Repeated-Dose 28-Day Oral Toxicity Study

#### 3.1.1. Behavioral, Physiological, and Macroscopic Examinations

Throughout the experimental period, no adverse behavioral, physiological or toxicological effects were observed in any group. Body weight increased progressively over time, as expected for normal growth, following similar patterns across all treatment groups (Figure 1B). Although male rats receiving *P. faecium* at 10^9^ CFU/day exhibited slightly lower body weights, this difference may be attributable to baseline differences (week 0) before treatment. Moreover, this effect was not observed in the group receiving the higher bacterial dose (10^10^ CFU/day) nor in females. Therefore, differences are attributed to inter-individual variability rather than the treatment. Consistently, food intake showed only a modest reduction in the *P. faecium* 10^9^ group of males (Figure 1C), consistent with their lower body weight.

The assessment of macroscopic changes in organs, such as the liver and spleen, did not reveal alterations in color, size, or morphology in any treatment group (Figure 1D,E).

#### 3.1.2. Translocation, Hematology and Biochemistry

The possible translocation of bacteria to the MLN and blood was evaluated by culturing samples in selective media under anaerobic conditions, followed by a specific PCR for their identification. These assessments did not identify colonies as *P. faecium* DSM 32890 or *B. longum* CECT 30763, confirming the absence of bacterial translocation and supporting the safety of the strains and doses administered orally.

In addition, hematological and biochemical analyses were performed to confirm the absence of systemic alterations. Both hemogram and serum biochemical profiles remained within physiological ranges, and no significant differences were observed between groups (Table 1). The only variation detected was a difference in serum phosphorus levels between the *B. longum* and placebo groups, unrelated to *P. faecium* administration.

#### 3.1.3. Intestinal Histology, Gut Barrier and Immunological Markers

Given that *P. faecium* DSM 32890 is a commensal bacterium that primarily could affect the gut, several parameters related to intestinal histology, immunity, and barrier integrity were evaluated to assess local safety.

**Figure 2:**
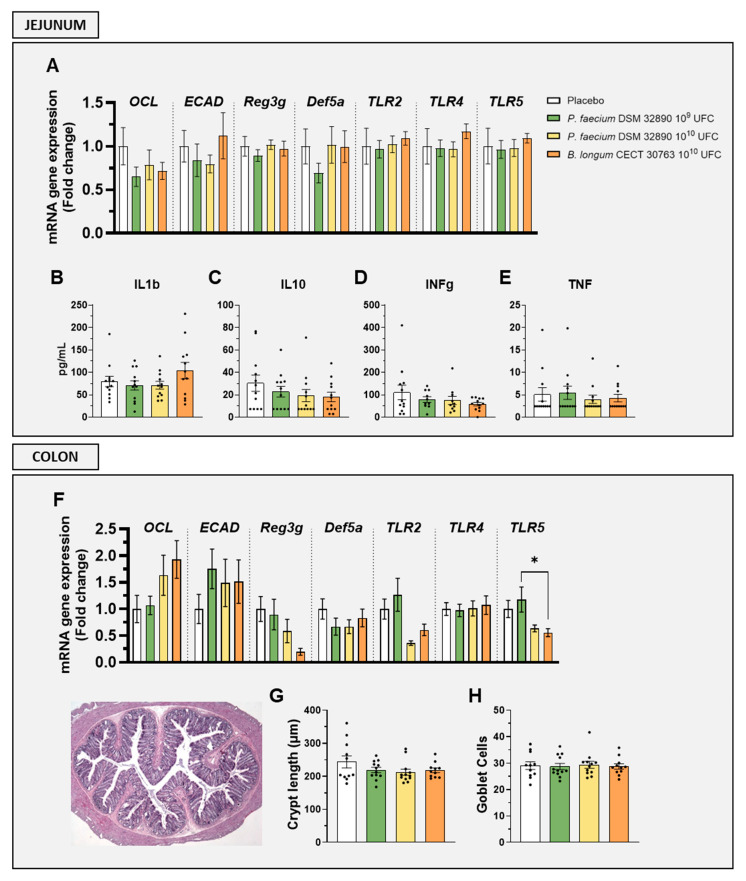
Evaluation of intestinal barrier integrity, immune activation, and histology. Gene expression by RT-qPCR analysis in (**A**) jejunum and (**F**) colon. Cytokine concentrations in jejunal tissue: (**B**) IL1b, (**C**) IL10, (**D**) IFNg, and (**E**) TNF. (**G**) Crypt length measurements and (**H**) mean goblet cell counts per crypt. Abbreviations: *OCL*, occludin; *ECAD*, E-cadherin; *Reg3g*, regenerating islet-derived protein 3 gamma; *Def5a*, alpha-defensin 5; *TLR*, Toll-like receptor; IL, interleukin; IFNg, interferon gamma; TNF, tumor necrosis factor. Data are expressed as mean ± SD. Differences were analysed by one-way ANOVA: * *p* < 0.05.

Gene expression analysis in the jejunum revealed that markers associated with gut permeability, such as occludin (*OCL*) and epithelial cadherin (*ECAD*), as well as epithelial defense (*Reg3g*, *Def5a*) and immune activation (*TLR2*, *TLR4*, and *TLR5*), were not altered by any treatment (Figure 2A). Similarly, jejunal concentrations of both pro-inflammatory (*IL1b*, *INFg* and *TNF*) and the anti-inflammatory cytokine (*IL-10*) remained unchanged following *P. faecium* administration (Figure 2B–E).

In the colon, the expression of genes *OCL*, *ECAD*, *Reg3g*, *Def5a*, *TLR2*, *TLR4*, and *TLR5* showed no significant differences between groups (Figure 2F).

Histological evaluation of colon tissue further confirmed the absence of structural alterations, as crypt length and goblet cell numbers were comparable across all treatments (Figure 2G,H).

**Figure 3:**
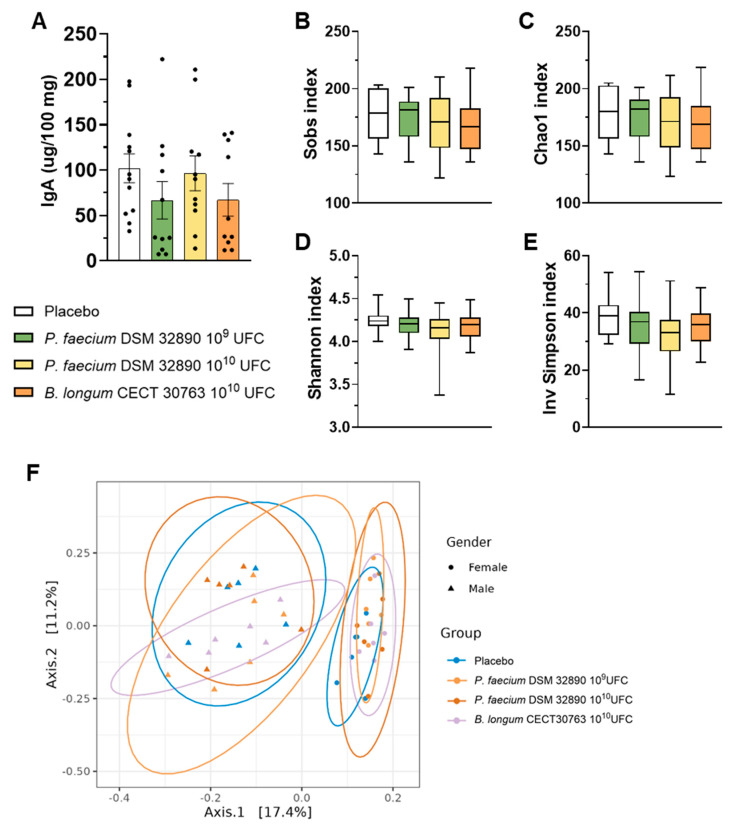
Fecal IgA concentration and microbiota analysis. (**A**) IgA concentration per 100 mg of rat feces. (**B**–**E**) Main alpha diversity indices of gut microbiota: Observed species, Chao1, Shannon, and Inverse Simpson. (**F**) Beta diversity grouped by sex. Data are expressed as mean ± SD.

In fecal samples, we assessed IgA concentrations, which did not differ among experimental groups (Figure 3A).

#### 3.1.4. Influence on Gut Microbiota

Analysis of 16S rRNA gene sequencing data performed in fecal samples revealed that any dose of *P. faecium* significantly affected the microbial richness or diversity in healthy rats (Figure 3B–E). Likewise, beta-diversity analysis showed clustering by sex but not by treatment (Figure 3F), indicating that administration of *P. faecium* DSM 32890 did not alter overall gut microbiota composition in healthy animals.

Differential abundance analysis identified 28 ASVs varying by treatment. Of these, 13 ASVs were identified in *B. longum* CECT 30763 group, 11 in *P. faecium* DSM 32890 10^9^ group, and 4 in *P. faecium* DSM 32890 1010 group (Supplementary Table S2). *B. longum*-treated group showed an increase in *B. longum* ASVs relative to the placebo (q < 10^−3^), accompanied by a significant depletion of Lachnospiraceae family ASVs (*Acetatifactor*, *Marvinbryantia* and certain members of the NK4A136 group; q < 0.05). *P. faecium* DSM 32890 treated groups showed altered abundances of the aforementioned Lachnospiraceae-related genus, and an additional depletion in *Alistipes* ASV (q < 0.04).

### 3.2. Safety and Tolerability in Humans

To evaluate the safety of consuming *P. faecium* DSM 32890, we conducted a first pilot short-term intervention in humans.

#### 3.2.1. Study Population Features

Descriptive characteristics of the study population are summarized in Table 2. The intervention trial included 20 participants: 9 with NW and 11 with OW. The mean age was approximately 40 years in both groups. Overall, 11 participants were female (4 in the NW group and 7 in the OW group), and the remaining were male. Most participants were Caucasian. The prevalence of comorbidities was higher among OW individuals, with 9 participants presenting at least one condition compared to 3 in the NW group (*p* = 0.0648). Hypertension was the most frequent comorbidity in the OW group (54.5%), whereas dyslipidemia was more common in the NW group (22.2%). Baseline gastrointestinal symptoms were significantly more frequent among OW participants (*p* = 0.0098). Thus, eight individuals (72.7%) reported at least one recurrent symptom, with constipation the most prevalent complaint. Finally, more than 60% of participants in the OW group took medication in the last month at baseline, compared with only 33% in the NW group.

Physical activity was evaluated using the short-form IPAQ questionnaire [11], and results were expressed as metabolic equivalent task (METs) units. As shown in Supplementary Figure S1A, participants in the NW group reported a higher weekly energy expenditure compared with those in the OW group. According to total METs per week, individuals were categorized as low, moderate, or high activity levels. Categorical analysis did not show statistically significant differences between the NW and OW groups. However, in the NW group, only one person had a low level of physical activity, whereas in the OW group, more participants had a low level of activity (Supplementary Figure S1B). Overall, physical activity remains essentially unchanged during the intervention period.

Self-perceived health was assessed using the PROMIS questionnaire, and t-scores were calculated according to official guidelines [10]. Both general physical health (GPH) and general mental health (GMH) scores tended to be slightly higher in the NW group, with a more pronounced difference observed in overall general health (GH). Consistent with the physical activity data, these self-reported health parameters remained unchanged, indicating that the bacterium supplementation did not influence participants’ perceived well-being (Supplementary Figure S1C).

As diet is a major determinant of gut microbiota composition and function, dietary intake was assessed using the validated PREDIMED food frequency questionnaire (FFQ) [12]. Overall, participants consumed higher amounts of fats and sugars, but lower amounts of carbohydrates, than the EFSA-recommended dietary guidelines [20]. No significant differences were observed in the intake of primary macronutrients either between NW and OW groups or between baseline and post-intervention time points, probably due to the small sample size (Supplementary Figure S1F,G). Even though fiber intake showed a non-significant trend toward higher consumption in the NW group, most of whom met the EFSA dietary recommendations for fiber [20]. In contrast, mean fiber intake in the OW group was below the recommended intake. Finally, analysis of the Healthy Eating Index (HEI) and the Dietary Inflammatory Index (DII) revealed no significant differences between groups; nonetheless, the OW group tended to follow a more pro-inflammatory dietary pattern according to DII values (Supplementary Figure S1D,E).

#### 3.2.2. Hematological and Biochemical Profiles in Humans

There were no differences in hemogram values, which remained stable after the intervention in both NW and OW participants. Only the mean MCHC showed a statistically significant difference between NW-posttreatment and OW-posttreatment groups (*p* = 0.04) (Supplementary Figure S2). However, the comparison within the OW group between pre- and post-treatment was not significant, suggesting that the observed change is unlikely to be attributable to the bacterium administration. Similarly, the proportions of leukocyte subpopulations did not differ significantly between baseline and post-probiotic administration in either group.

Biochemical analyses included assessments of lipid profile (triglycerides, total cholesterol, LDL cholesterol, HDL cholesterol, and non-HDL cholesterol), renal function (urea, creatinine, and uric acid), hepatic function (total protein, AST, and ALT), and glucose homeostasis (glucose, insulin, HbA1c, and HOMA-IR). As shown in Supplementary Figure S3, glycemia, insulin, and HOMA-IR differed significantly between the NW and OW groups at both time points, as expected. Triglyceride levels were significantly higher in the OW group post-treatment compared to the NW group; however, as with MCHC, the increases were not significant when comparing pre- and post-treatment values within the OW group.

Collectively, these results indicate that daily supplementation with *P. faecium* DSM 32890 does not produce systemic effects detectable through hematological or biochemical markers in the short term.

#### 3.2.3. Bowel Habits and Gastrointestinal Symptoms

**Figure 4:**
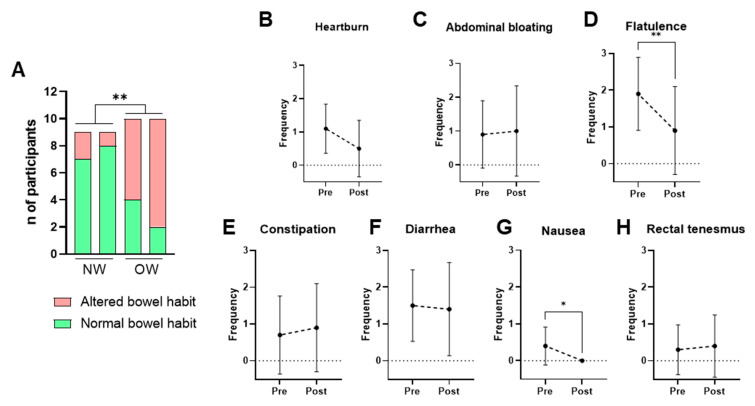
Self-reported intestinal habits and gastrointestinal symptoms. (**A**) Bowel habit in NW and OW participants before and after the intervention. (**B**–**H**) Frequency per week of gastrointestinal symptoms (0–3 scale) in OW participants pre- and post-treatment. Data are presented as mean ± SD. Differences were analyzed by paired *t*-test, * *p* < 0.05, ** *p* < 0.01.

Bowel habits were assessed using the weekly bowel movement frequency and the stool consistency according to the Bristol Stool Scale. A normal bowel habit was defined as a frequency of 4–14 defecations per week and a Bristol score between 3 and 5, while all other cases were classified as altered bowel habits. The NW group consistently exhibited healthier bowel patterns than the OW group across all time points. Bacterium supplementation did not significantly alter these parameters in any of the groups (Figure 4A).

Given that the OW group represents the target population, self-reported gastrointestinal symptoms were examined further among these participants. The results showed that consumption of *P. faecium* DSM 32890 was well tolerated and even associated with improvements in specific symptoms (Figure 4B–H). After the intervention period, significant reductions in flatulence (Figure 4D) and nausea (Figure 4G) were observed relative to baseline. Overall, both the frequency and intensity of gastrointestinal complaints were low, supporting the strain’s good gastrointestinal tolerability.

#### 3.2.4. Effects on Body Composition

**Figure 5:**
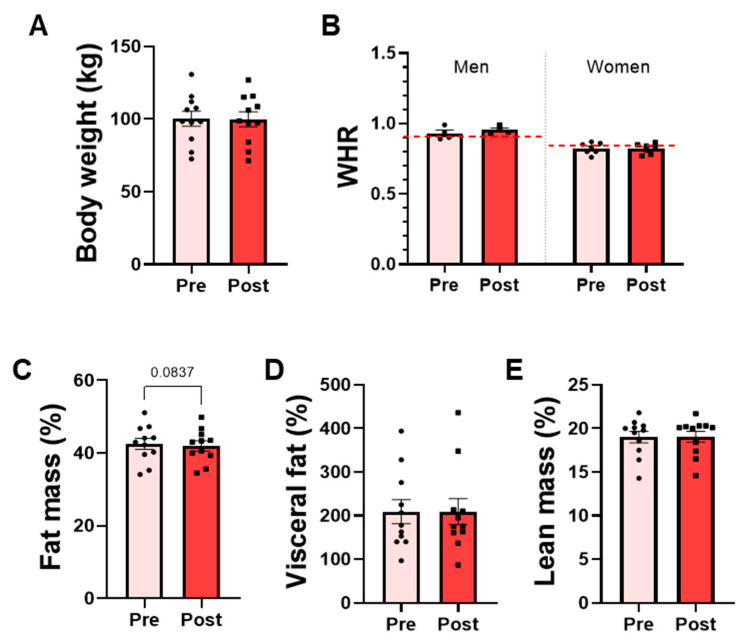
Anthropometric and body composition parameters in overweight participants. (**A**) Body weight measured by scale. (**B**) Waist-to-hip ratio (WHR) in men and women. The red dotted lines represent the WHO-recommended cut-off points for men and women. DEXA results: (**C**) Total fat mass percentage, (**D**) visceral fat percentage, and (**E**) lean mass percentage. Data are presented as mean ± SD. Paired *t*-test.

The effects on body composition indexes were analyzed before and after the intervention in the OW group, which represents the target population for future efficacy trials. No significant differences were detected in body weight or body composition variables following the bacterial supplementation. Mean body weight remained stable throughout the intervention, and the waist-to-hip ratio (WHR), used to assess body fat distribution, did not change in either men or women (Figure 5A,B). Similarly, lean mass and visceral fat percentages did not differ significantly between pre- and post-intervention (Figure 5D,E). A modest trend toward reduced total fat mass percentage was observed associated with *P. faecium* DSM 32890 (paired *t*-test, *p* = 0.0837) (Figure 5C).

## 4. Discussion

The interest in exploring the potential use of indigenous intestinal bacteria linked to human health and preclinically proven to be effective in managing non-communicable disorders has increased exponentially in the last decade [21]. Notwithstanding, the path toward developing microbiome-based products also requires safety evaluations, as there is no history of use of these bacteria for human consumption. This is the case for the commensal bacterial species *P. faecium*, which does not have GRAS or QPS status, and further development as a next-generation probiotic or live biotherapeutic product requires a safety evaluation. Accordingly, we demonstrated, through both preclinical and clinical trials, that *P. faecium* DSM 32890 is safe and well-tolerated with no evidence of adverse effects. The species *P. faecium* is a commensal bacterium that naturally inhabits the human gut, where it ferments succinate and produces SCFAs, such as propionate, which may contribute to intestinal homeostasis and health maintenance [22,23]. Our group has previously shown that the prevalence of this species is higher in non-obese subjects than in overweight or obese subjects, and that it exerts specific beneficial effects on immune function and body weight regulation in obesity models [4], highlighting its potential as a next-generation probiotic. The present work aims to advance this path by confirming the safety of the sub-chronic consumption of *P. faecium* DSM 32890 in an animal model and its tolerability in humans. Importantly, no metabolic effects were observed in the animal study, as expected in healthy rats. Given the study’s safety-focused design and the absence of metabolic dysfunction in the model, the detection of metabolic or inflammatory improvements was neither expected nor within the scope of the present work.

The oral sub-chronic toxicity study showed that rats treated with a high dose of *P. faecium* DSM 32890 (10^10^ CFU/day) exhibited no adverse effects, behavioral changes, or alterations in any health-related parameters. The potential for bacteremia or bacterial translocation represents an essential indicator of pathogenicity and systemic infection risk. Accordingly, we also evaluated, using PCR analyses with strain-specific primers, that the administered strain was not translocated to MLNs or blood. Although a few bacterial colonies were detected on culture plates, these were most likely due to cross-contamination, as previously reported in other oral toxicity studies [8,9,24], since none were identified as the administered bacterial species. Also, similar counts were detected in control samples.

To further confirm the absence of systemic toxicity, biochemical, hematological, and inflammatory markers were assessed. Kidney function parameters, including urea and creatinine, remained within normal ranges and were not altered by *P. faecium* administration. Similarly, hepatic enzyme activities (ALT and ALP) were within physiological limits, indicating normal liver function. Glucose and lipid metabolism were also unaffected. These findings were consistent across both sexes.

Given that *P. faecium* did not translocate to MLNs or the bloodstream, we next examined potential local effects within the intestine. In both the jejunum and the colon, no signs of inflammation or epithelial damage were detected. Gut barrier integrity markers (*Ocl* and *Ecad*) and histological evaluations revealed no alterations, and the expression of proinflammatory cytokines, the release of IgA into the lumen, and antimicrobial peptides (*Reg3g* and *Def5a*) remained unaffected. Altogether, these results indicate the absence of deleterious interactions between *P. faecium* DSM 32890 and the intestinal immune system. In line with these findings, previous studies have reported reduced abundance of *P. faecium* under conditions associated with increased inflammation and impaired intestinal barrier function [4,25,26]. This not only reinforces the lack of pathological immune activation observed here but also supports the notion that *P. faecium* may exert a protective role in maintaining intestinal homeostasis.

The analysis of fecal microbiota revealed that neither the richness and alpha-diversity indices (Observed species, Chao1, Shannon, or Inverse Simpson) nor the beta-diversity metrics differed significantly between *P. faecium*-treated and placebo animals. While previous studies have reported shifts in microbial composition in obese mice [4], our experiment was conducted in healthy rats, whose gut microbiota is known to be more stable and resilient to external modulation [27,28]. This physiological robustness likely explains the absence of detectable changes in microbial diversity following the bacterial administration. Interestingly, beta-diversity analyses indicated that sex was the main driver of microbial variation, suggesting that sex hormones exert a substantial influence on gut microbiota structure under healthy conditions and that this is an important variable to consider in microbiome research [29].

Differences in the abundance of certain bacterial taxa detected in the *P. faecium*-treated mouse group suggest that the administered strain may, to some extent, affect gut ecology. For instance, it affected the abundance of taxa in the Lachnospiraceae NK4A136 group, as observed with *B. longum* administration. However, a more precise taxonomic resolution would be needed to interpret the possible biological consequences of these changes. Unlike the enrichment of *B. longum* detected in the group receiving this species, no *P. faecium*-related ASVs were detected after prevalence filtering. This could reflect the generally low levels of colonization by *P. faecium* DSM 32890, a strain and a species primarily identified in the human commensal microbiota [2]. Moreover, short-amplicon 16S rRNA gene sequencing data are insufficient to precisely identify taxa at species and strain levels. To address these limitations, alternative methods, such as shotgun metagenomics, are recommended for future studies to elucidate colonization and the possible engraftment of *P. faecium* DSM 32890-treated subjects.

Collectively, the preclinical findings demonstrate that *P. faecium* DSM 32890 is a safe bacterial strain for repeated consumption. Based on this safety profile in rats, a subsequent pilot clinical trial was designed to evaluate its tolerability in healthy and overweight human participants.

In the clinical trial, both NW and OW individuals were included to evaluate the tolerability of *P. faecium* DSM 32890 in healthy participants and in the target population. This design also enabled us to assess whether tolerability differed according to metabolic status. The study population was well balanced with respect to age and sex. Treatment adherence was excellent, with no participants dropping out of the trial, and capsule intake compliance was high. Importantly, no relevant adverse effects were reported throughout the intervention period.

The intervention did not induce significant changes in hematological or biochemical parameters. Only MCHC and triglyceride levels differed significantly between NW and OW participants at post-treatment. However, within-group comparisons in the OW cohort revealed no significant pre- to post-treatment differences, suggesting that these variations were not attributable to the bacterium intake. Previous studies have reported that probiotic supplementation may enhance iron absorption and, consequently, increase hemoglobin concentrations by increasing SCFA production and reducing intestinal pH [30]. Nevertheless, the duration of our intervention was shorter and likely insufficient to elicit such effects. The absence of alterations in liver enzymes and kidney function markers further supports the safety profile of *P. faecium* DSM 32890. This finding is particularly relevant given that individuals with obesity and type 2 diabetes frequently present hepatic steatosis, which can compromise liver function [31].

Although some probiotics have been used to alleviate gastrointestinal symptoms [32,33], they may also occasionally cause mild gastrointestinal discomfort, such as bloating, gas, or abdominal cramps, at the beginning of treatment [34,35]. In our study, *P. faecium* supplementation did not alter bowel habits in either group, but it significantly improved specific gastrointestinal symptoms, including flatulence and nausea, in OW participants. These results contrast with the recent meta-analysis by Soltani et al. [36], which examined 32 clinical trials evaluating the effects of probiotic supplementation in individuals with type 2 diabetes and reported a relatively high frequency of gastrointestinal discomfort associated with probiotic intake. The favorable gastrointestinal tolerance profile, together with observed symptom-specific improvement, suggests that *P. faecium* DSM 32890 may offer advantages in this regard. However, it is important to note that this study was conducted in adults without known gastrointestinal disease. Therefore, the present tolerability findings should be interpreted with caution when considering populations with intestinal inflammatory conditions and/or barrier dysfunction. In particular, although probiotic bacteria are increasingly used by patients with gastrointestinal disorders [37], the safety profile and immune effects of *P. faecium* DSM 32890 may differ substantially in the context of severe inflammatory enteropathies, such as ulcerative jejunitis, celiac crisis, or refractory celiac disease. In these conditions, extrapolation from the current data would be inappropriate without dedicated, disease-specific safety and efficacy studies.

Finally, despite the short duration of the study (15 days) and the small sample size (N = 11), we conducted an exploratory body composition analysis in OW participants; however, no statistically significant differences were observed. However, a non-significant trend towards reduced total fat mass percentage (*p* ≈ 0.08) was detected. The lack of significant effects is likely due to the short duration of the intervention and the small sample size, which represent the study’s major limitations, as it would typically require 4–26 weeks [36]. Overall, the observed efficacy trends should be considered as future hypothesis to design longer and adequately powered intervention studies to robustly assess the potential metabolic or inflammatory effects of *P. faecium* DSM 32890.

## 5. Conclusions

This study provides the first evidence supporting the safety of a specific strain of the commensal species *P. faecium* for oral consumption. Repeated oral administration of *P. faecium* DSM 32890 exhibited a safety and tolerability profile, with no signs of toxicity, adverse outcomes, or immune and biochemical alterations in either rats or humans. These findings provide a basis for larger-scale, longer-duration interventions to assess the potential long-term benefits for inflammation, body composition, and energy metabolism.

## Figures and Tables

**Table 1:** Hematological and biochemical parameters of the toxicity study in rats.

	Placebo	*P. faecium* 10^9^	*P. faecium* 10^10^	*B. longum* 10^10^	*p*-Value
WBC (10^9^/L)	12.24 ± 3.18	13.78 ± 5.1	13.18 ± 4.98	14.76 ± 3.83	ns
Neu # (10^9^/L)	1.72 ± 0.81	1.78 ± 0.63	1.58 ± 0.96	1.88 ± 0.96	ns
Lym # (10^9^/L)	9.66 ± 2.18	11.18 ± 4.35	10.69 ± 4.13	12.03 ± 2.83	ns
Mon # (10^9^/L)	0.68 ± 0.36	0.65 ± 0.38	0.72 ± 0.44	0.66 ± 0.25	ns
Eos # (10^9^/L)	0.16 ± 0.1	0.15 ± 0.08	0.16 ± 0.08	0.15 ± 0.06	ns
Bas # (10^9^/L)	0.02 ± 0.02	0.03 ± 0.02	0.03 ± 0.02	0.03 ± 0.02	ns
Neu % (%)	13.42 ± 3.95	13.5 ± 3.32	11.84 ± 4.93	12.27 ± 3.64	ns
Lym % (%)	79.95 ± 5.99	80.72 ± 4.39	81.61 ± 7.06	82.08 ± 4.12	ns
Mon % (%)	5.26 ± 1.88	4.59 ± 1.87	5.22 ± 2.07	4.46 ± 1.03	ns
Eos % (%)	1.21 ± 0.52	1.03 ± 0.4	1.14 ± 0.41	1.02 ± 0.39	ns
Bas % (%)	0.17 ± 0.11	0.17 ± 0.07	0.19 ± 0.1	0.18 ± 0.08	ns
RBC (10^12^/L)	8.02 ± 1.85	8.78 ± 1.95	8.51 ± 1.91	8.85 ± 0.51	ns
HGB (g/L)	15.12 ± 3.36	16.58 ± 3.62	16.48 ± 3.59	17.24 ± 1	ns
HCT (%)	40.61 ± 8.89	44.59 ± 9.76	44.2 ± 9.71	46.08 ± 2.55	ns
MCV (fL)	50.88 ± 1.92	50.96 ± 1.79	52.05 ± 1.29	52.11 ± 1.32	ns
MCH (pg)	18.93 ± 0.78	18.96 ± 0.97	19.38 ± 0.42	19.5 ± 0.55	ns
MCHC (g/dL)	37.15 ± 0.75	37.23 ± 0.73	37.3 ± 0.84	37.44 ± 0.7	ns
RDW-CV (%)	12.01 ± 0.51	12.4 ± 0.63	12.17 ± 0.52	12.18 ± 0.62	ns
PLT (10^9^/L)	501.58 ± 304.79	682.67 ± 278.9	835 ± 325.41	536.42 ± 336.4	ns
MPV (fL)	6.93 ± 0.66	6.72 ± 0.43	6.44 ± 0.33	6.47 ± 0.45	ns
A/G	1.68 ± 0.27	1.76 ± 0.42	1.58 ± 0.18	1.66 ± 0.19	ns
B/C	30.12 ± 4.71	26.36 ± 3.39	30.18 ± 7.26	30.33 ± 3.49	ns
C-Ca (mg/dL)	11.43 ± 0.37	11.18 ± 1.19	11.58 ± 0.36	11.83 ± 0.22	ns
GLOB (g/dL)	2.42 ± 0.3	2.41 ± 0.6	2.46 ± 0.27	2.53 ± 0.21	ns
UREA (mg/dL)	37.45 ± 3.94	34.62 ± 5.46	40.37 ± 11.38	38.08 ± 4.77	ns
ALB (g/dL)	3.95 ± 0.22	3.99 ± 0.41	3.95 ± 0.33	4.14 ± 0.33	ns
ALP (U/L)	187.92 ± 53.58	171.75 ± 25.11	173.33 ± 48.37	169.17 ± 35.27	ns
ALT (U/L)	39.67 ± 7.25	42.58 ± 10.99	34.58 ± 5.21	37.17 ± 6.28	ns
AMY (U/L)	312.25 ± 31.97	310.83 ± 34.86	315.67 ± 34.54	315.08 ± 31.48	ns
BUN (mg/dL)	17.52 ± 1.87	16.17 ± 2.55	18.88 ± 5.36	17.8 ± 2.22	ns
CHOL (mg/dL)	91.17 ± 23.24	97.25 ± 17.07	102.17 ± 18.95	95.75 ± 12.59	ns
CREA (mg/dL)	0.58 ± 0.06	0.59 ± 0.08	0.62 ± 0.06	0.59 ± 0.06	ns
GLU (mg/dL)	197 ± 24.62	188.33 ± 33.79	207.75 ± 27.46	226.17 ± 57.07	ns
LIPA (U/L)	28.5 ± 2.15	29 ± 1.76	28.42 ± 1.73	28 ± 1.21	ns
PHOS (mg/dL)	6.23 ± 0.83	6.96 ± 1.01	6.91 ± 0.95	8.2 ± 1.54	*
TBIL (mg/dL)	0.04 ± 0.1	0.05 ± 0.1	0 ± 0	0.04 ± 0.07	ns
TP (g/dL)	6.37 ± 0.28	6.36 ± 0.89	6.55 ± 0.22	6.68 ± 0.37	ns

Data are expressed as mean ± SD. * *p* < 0.05 *B. longum* vs. placebo. ns: not significant. #: content.

**Table 2:** Baseline characteristics of participants of the clinical trial.

	Total Population (N = 20)	Normal Weight (NW) (N = 9)	Overweight (OW) (N = 11)	*p*-Value
Age	39.90 ± 14.03	38.89 ± 11.47	40.73 ± 16.34	0.7716
Female	11 (55.0%)	4 (44.4%)	7 (63.6%)	>0.9999
Weight (kg)	84.97 ± 22.73	66.00 ± 10.16	100.48 ± 17.63	<0.0001
Height (cm)	170.55 ± 6.67	174.67 ± 6.32	167.18 ± 5.21	0.0119
BMI (kg/m^2^)	29.43 ± 8.53	21.53 ± 2.16	35.89 ± 5.70	<0.0001
Smokers	7 (35.0%)	3 (33.3%)	4 (36.4%)	>0.9999
Ethnicity				
Caucasian	19 (95.0%)	9 (100%)	10 (90.9%)	>0.9999
Other	1 (5.0%)	0	1 (9.1%)	
Diseases (presence or absence)				0.0648
Hypertension	6 (30.0%)	0	6 (54.5%)	0.0141
Dyslipidemia	3 (15.0%)	2 (22.2%)	1 (9.1%)	0.5658
Headache	3 (15.0%)	1 (11.1%)	2 (18.2%)	>0.9999
Hormonal disorders	2 (10.0%)	0	2 (18.2%)	0.4789
Others	3 (15.0%)	0	3 (27.3%)	0.4789
Gastrointestinal symptoms				
Vomit	0	0	0	>0.9999
Diarrhoea	4 (20.0%)	1 (11.1%)	3 (27.3%)	0.5913
Constipation	4 (20.0%)	0	4 (36.4%)	0.0941
Stomach pain	2 (10.0%)	1 (11.1%)	1 (9.1%)	>0.9999
None	11 (55.0%)	8 (88.9%)	3 (27.3%)	0.0098
Surgeries or hospitalizations	12 (60.0%)	3 (33.3%)	9 (81.8%)	0.0648
Medication intake in the last month	10 (50.0%)	3 (33.3%)	7 (63.6%)	0.3698

Data are expressed as mean ± SD for continuous variables and as n (%) for categorical variables. Group comparisons were performed using the *t*-test and Chi-square (χ^2^) test, respectively. BMI: body mass index.

## Data Availability

Original data available upon request.

## Supplementary Materials

The following supporting information can be downloaded at: https://www.mdpi.com/article/10.3390/nu18030498/s1, Supplementary Table S1. Primer sequences used for PCR and qPCR analyses. Supplementary Table S2. Differentially abundant taxa/species found in the treatment groups compared to placebo. Supplementary Figure S1. Physical activity, perceived health, and dietary assessment results. Supplementary Figure S2. Hemogram results from the clinical trial. Supplementary Figure S3. Biochemical blood analysis results from the clinical trial.

## Abbreviations

The following abbreviations are used in this manuscript:

ALP	Aspartate aminotransferase
ALT	Alanine aminotransferase
BMI	Body mass index
CFUs	Colony-forming units
CSIC	Spanish National Research Council
Def5a	Alpha-Defensin 5
DII	Dietary Inflammatory Index
ECAD	Epithelial cadherin
Ecad	E-cadherin
GH	General Health
GMH	General Mental Health
GPH	General Physical Health
HbA1c	Glycated hemoglobin
HEI	Healthy Eating Index
INFg	Interferon γ
MCHC	Mean Corpuscular Hemoglobin Concentration
METs	Metabolic Equivalent Task units
MLNs	Mesenteric lymph nodes
NW	Normal weight
OCL	Occludin
OW	Overweight
PBS	Phosphate-buffered saline
Reg3g	Regenerating islet-derived protein 3 gamma
SCFA	Short chain fatty acid
SD	Standard deviation
TNFa	Tumor necrosis factor α
TSS	Total Sum Scaling
WHR	Waist-to-Hip Ratio
