# Supplementary material for: Safety and Tolerability of the Gut Bacterium Phascolarctobacterium faecium DSM 32890
Source: Nutrients. 2026 Feb 2;18(3):498. doi: 10.3390/nu18030498 (PMC12899801; doi:10.3390/nu18030498)
Supplement: Supplementary file 1 [file nutrients-18-00498-s001.zip › nutrients-4087023-supplementary.pdf]

Supplemental figures

Supplementary Table S1. Primer sequences used for PCR and qPCR analyses.

Gene	Sequence	
α DEF 5	F	ACTTGTCCTCCTTTCTGCCC
	R	ATCCCCATAATGCCTTCTCC
TLR5	F	GGGCAGCAGAAAGACGGTAT
	R	CAGGCACCAGCCATCTTAA
OCLN	F	CTACTCCTCCAACGGCAAAG
	R	AGTCATCCACGGACAAGGTC
TLR2	F	CTCCTGTGAACTCCTGTCCTT
	R	AGCTGTCTGGCCAGTCAAC
ECADH	F	CTGGACCGAGAGAGTTACCC
	R	GGCACCGACCTCATTCTCAA
TLR4	F	TGCTACAGTTCATCTGGGTTTCTG
	R	CTGTGAGGTCGTTGAGGTTAGAAG
Reg3γ	F	CTGTCTCTGGAAGTCACTGTGG
	R	GGGTTCTTGATTTGGTGAGG
RpLp2	F	TCGCTCAGGGTGTTGGCAAG
	R	AGGCCAAATCCCATGTCGTC
P. faecium DSM 32890	F	GGCGGCTTAATAAGTCGAGC
	R	CGTTCGCTACCCTGGCTTTC
BifiThrS	F	AAGGACGGCTTCTACTACGA
	R	AAGATCAGGTTGTGCATCGG

Supplementary Table S2. Differentially abundant species found in the treatment groups compared to Placebo.

ASV	Kingdom	Phylum	Class	Order	Family	Genus	Species	Treatment	coef	stderr	pval	qval
ASV179	Bacteria	Firmicutes	Clostridia	Lachnospirales	Lachnospiraceae	Acetatifactor		B. longum 10^10	-2,70	0,48	1,49E-06	6,95E-05
ASV436	Bacteria	Actinobacteriota	Actinobacteria	Bifidobacteriales	Bifidobacteriaceae	Bifidobacterium	longum	B. longum 10^10	2,32	0,42	1,93E-06	8,73E-05
ASV179	Bacteria	Firmicutes	Clostridia	Lachnospirales	Lachnospiraceae	Acetatifactor		P. faecium 10^9	-2,49	0,48	6,41E-06	2,32E-04
ASV	Bacteria	Firmicutes	Clostridia	Lachnospirales	Lachnospiraceae	Acetatifactor		P. faecium	-2,41	0,48	1,13E	3,54E

179								10^10			-05	-04
ASV260	Bacteria	Firmicutes	Clostridia	Lachnospirales	Lachnospiraceae	Marvinbryantia		P. faecium 10^10	-1,79	0,44	1,87E-04	4,35E-03
ASV260	Bacteria	Firmicutes	Clostridia	Lachnospirales	Lachnospiraceae	Marvinbryantia		P. faecium 10^9	-1,78	0,44	1,95E-04	4,47E-03
ASV260	Bacteria	Firmicutes	Clostridia	Lachnospirales	Lachnospiraceae	Marvinbryantia		B. longum 10^10	-1,75	0,44	2,41E-04	5,18E-03
ASV88	Bacteria	Firmicutes	Clostridia	Lachnospirales	Lachnospiraceae			B. longum 10^10	-1,62	0,41	2,59E-04	5,42E-03
ASV224	Bacteria	Firmicutes	Clostridia	Lachnospirales	Lachnospiraceae			B. longum 10^10	-2,35	0,59	2,81E-04	5,81E-03
ASV81	Bacteria	Firmicutes	Clostridia	Lachnospirales	Lachnospiraceae	Lachnospiraceae NK4A136 group		B. longum 10^10	-2,79	0,71	3,09E-04	6,12E-03
ASV78	Bacteria	Firmicutes	Clostridia	Lachnospirales	Lachnospiraceae	Lachnospiraceae NK4A136 group		P. faecium 10^9	2,78	0,71	3,37E-04	6,41E-03
ASV446	Bacteria	Firmicutes	Clostridia	Oscillospirales	Oscillospiraceae	NK4A214 group		P. faecium 10^10	-1,14	0,30	4,33E-04	8,01E-03
ASV162	Bacteria	Firmicutes	Clostridia	Lachnospirales	Lachnospiraceae	Lachnospiraceae NK4A136 group		P. faecium 10^9	1,72	0,45	4,48E-04	8,19E-03
ASV	Bacteria	Firmicutes	Clostridia	Oscillospirales	Ruminococcaceae	[Eubacterium] siraeum group		P. faecium 10^9	-1,96	0,52	4,63E-04	8,36E-03

261												
ASV 115	Bacteria	Firmicutes	Clostridia	Lachnospirales	Lachnospiraceae	Lachnospiraceae NK4A136 group		P. faecium 10^9	2,09	0,57	7,10E-04	1,19E-02
ASV 261	Bacteria	Firmicutes	Clostridia	Oscillospirales	Ruminococcaceae	[Eubacterium] siraeum group		B. longum 10^10	-1,77	0,52	1,33E-03	1,94E-02
ASV 95	Bacteria	Bacteroidota	Bacteroidia	Bacteroidales	Muribaculaceae			P. faecium 10^9	1,90	0,56	1,48E-03	2,05E-02
ASV 107	Bacteria	Firmicutes	Clostridia	Lachnospirales	Lachnospiraceae	Lachnospiraceae UCG-001		B. longum 10^10	-1,30	0,38	1,50E-03	2,05E-02
ASV 83	Bacteria	Firmicutes	Clostridia	Oscillospirales	[Eubacterium] coprostanoligenes group			B. longum 10^10	2,30	0,70	2,16E-03	2,66E-02
ASV 238	Bacteria	Firmicutes	Clostridia	Lachnospirales	Lachnospiraceae	A2		P. faecium 10^9	1,27	0,39	2,14E-03	2,66E-02
ASV 246	Bacteria	Bacteroidota	Bacteroidia	Bacteroidales	Muribaculaceae			P. faecium 10^10	-1,40	0,43	2,15E-03	2,66E-02
ASV 81	Bacteria	Firmicutes	Clostridia	Lachnospirales	Lachnospiraceae	Lachnospiraceae NK4A136 group		P. faecium 10^9	-2,29	0,71	2,46E-03	2,91E-02
ASV 111	Bacteria	Cyanobacteria	Vampirivibrionia	Gastranaerophilales				B. longum 10^10	-1,96	0,61	2,57E-03	2,99E-02
ASV 446	Bacteria	Firmicutes	Clostridia	Oscillospirales	Oscillospiraceae	NK4A214 group		B. longum 10^10	-0,95	0,30	2,78E-03	3,11E-02

ASV56	Bacteria	Firmicutes	Clostridia	Lachnospirales	Lachnospiraceae	Lachnospiraceae NK4A136 group		B. longum 10^10	2,74	0,87	2,89E-03	3,21E-02
ASV389	Bacteria	Firmicutes	Clostridia	Lachnospirales	Lachnospiraceae			P. faecium 10^9	-0,95	0,31	3,64E-03	3,85E-02
ASV61	Bacteria	Bacteroidota	Bacteroidia	Bacteroidales	Rikenellaceae	Alistipes		P. faecium 10^9	-1,34	0,44	4,18E-03	4,26E-02
ASV359	Bacteria	Firmicutes	Clostridia	Oscillospirales	Ruminococcaceae			B. longum 10^10	1,00	0,33	4,43E-03	4,47E-02

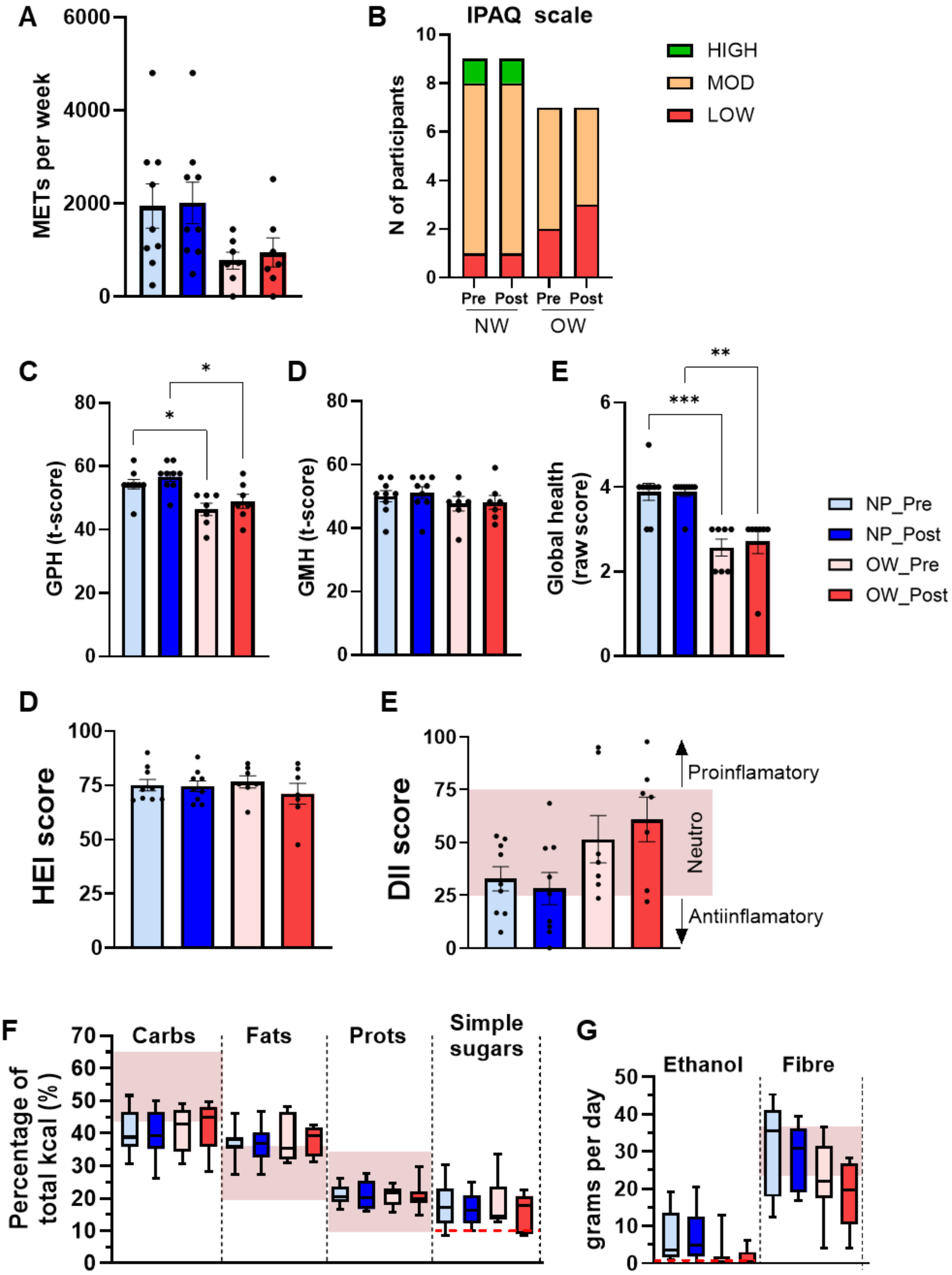

Supplementary Figure S1. Results of physical activity, perceived health, and dietary assessment results. (A) Weekly metabolic equivalent task (MET) values and (B) categorical classification according to the IPAQ questionnaire scale. (C) PROMIS t-score results for global physical health (GPH), global mental health (GMH), and global health (GH). (D–E) Dietary indices: Healthy Eating Index (HEI) and Dietary Inflammatory Index (DII). (F–G) Macronutrient intake (carbohydrates, fats, proteins, simple sugars, ethanol, and fiber). Shaded areas indicate the adequate daily intake range according to EFSA recommendations. The red dotted line marks the upper limit for daily sugar or ethanol intake recommended by EFSA. Data are presented as mean ± SD.

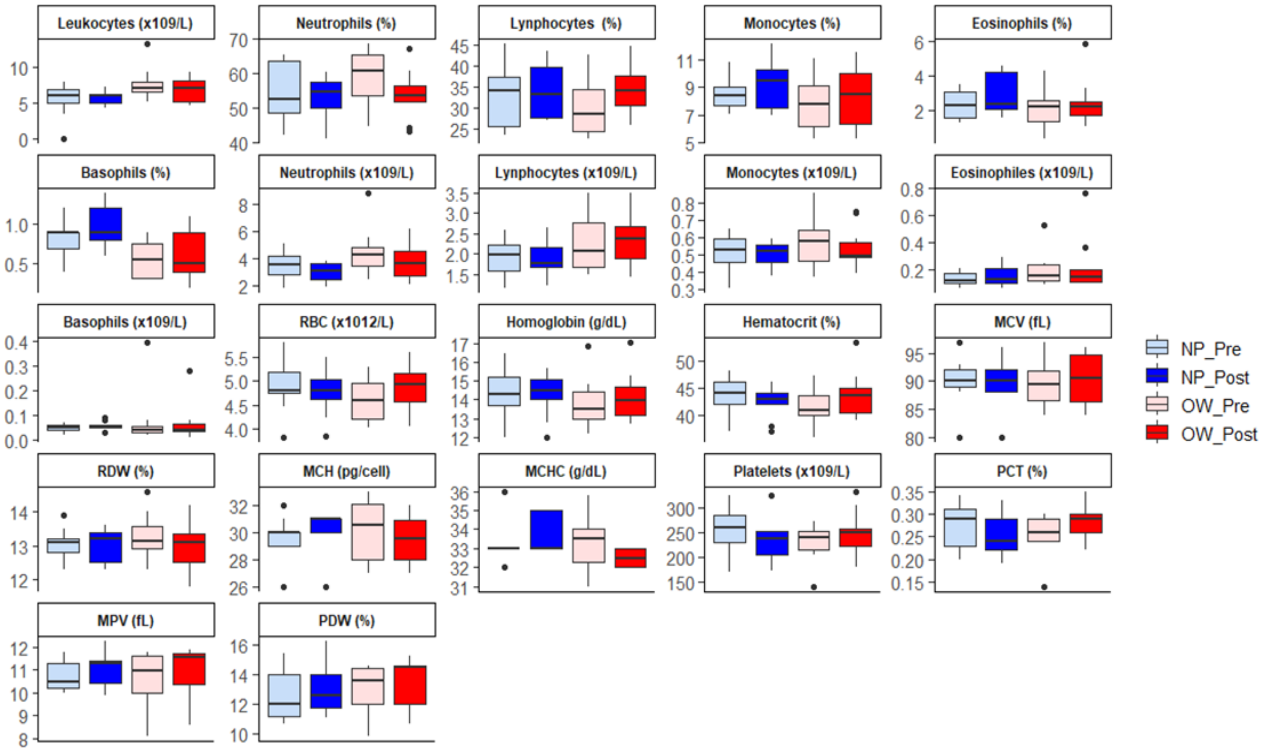

Parameter	Group 1	Group 2	p-value
MCHC (g/dL)	NP_Post	OW_Post	0,0403

Supplementary Figure S2. Hemogram results from the intervention trial in humans. Abbreviations: RBC, red blood cells; MCV, mean corpuscular volume; RDW, red cell distribution width; MCH, mean corpuscular hemoglobin; MCHC, mean corpuscular hemoglobin concentration; PCT, plateletcrit; MPV, mean platelet volume; PDW, platelet distribution width. Data are presented as mean ± SD. Differences were analyzed using the Kruskal–Wallis test, and those statistically significant are indicated in the table together with the corresponding adjusted p-values.

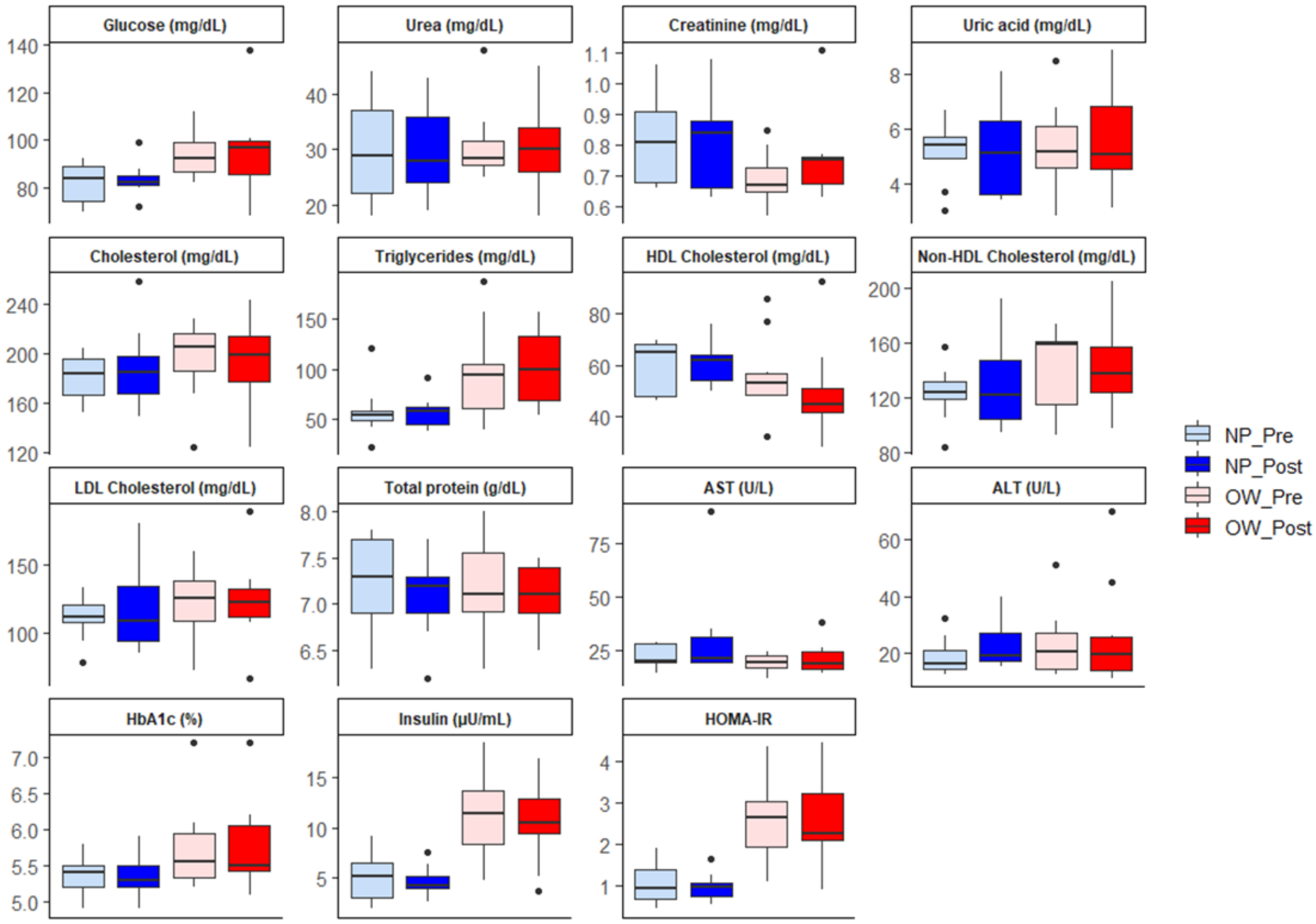

Parameter	Group 1	Group 2	p-value
Glucose (mg/dL)	NP_Pre	OW_Pre	0,0775
	NP_Post	OW_Pre	0,0957
Triglycerides (mg/dL)	NP_Post	OW_Post	0,0517
Insulin (μU/mL)	NP_Pre	OW_Pre	0,0076
	NP_Post	OW_Post	0,0151
HOMA-IR	NP_Pre	OW_Pre	0,0045
	NP_Post	OW_Post	0,0093

Supplementary Figure S3. Biochemical blood analysis results from the clinical trial. Abbreviations: AST, aspartate aminotransferase; ALT, alanine aminotransferase; HbA1c, glycated hemoglobin. All parameters were analyzed using the Kruskal–Wallis test, and significant differences are indicated in the table together with the corresponding adjusted p-values. Data are presented as mean ± SD.
